# Twenty-Eighth Annual Commencement of the Buffalo Medical College

**Published:** 1874-02

**Authors:** 


### Editorial.

### Twenty-Eighth Annual Commencement of the Buffalo Medical College.

The twenty-eighth annual commencement exercise of the Buffalo Medical College was held at St James Hall Tuesday evening Feb 24th, at which time the degree of Doctor of Medicine was conferred upon thirty-six graduates. After music by Poppenberg's orchestra, the exercises were opened with prayer by Rev. D. R. Fraser. Dr. Miner, Dean of the Faculty, then read the following list of graduates upon whom the Council, upon recommendation of the Faculty and Curators, had voted to confer the Degree of Doctor of Medicine:

John R. Selover, Bath, N. Y.; Edward Nathaniel Brush, Buffalo, N. Y.; Edward Mott Moore, Jr., A. B., Rochester, N. Y.; William Ellsworth Fitzgibbons, Hornellsville, N. Y.; Bernard Bartow, Buffalo, N. Y.; John

Andrew Pettit, Buffalo, N. Y.; Jacob Depew Terwilliger, High Falls, N. Y.; John Preston Frink, Buffalo, N. Y.; George Matthew Blake, Dansville, N. Y.; Elroy Sabin West, Buffalo, N. Y.; George Morrison Gillett, Cuba, N. Y.; William Judson Howe, Buffalo, N. Y.; William Davis Whitney, Youngsville, Pa.; John Wockener, Bennington, N. Y.; Theophilus Henry Boysen, Buffalo, N. Y.; Thomas Lawrence Barry, Buffalo, N. Y.; Robert Porter Bush, Penn Yan, N. Y.; Paul Alexander Quick, Sugar Run, Pa.; James Warren Graham, Hubbard, Ohio; Darius Gilead Pickett, Stockton, N. Y.; John M. Folts, Wayland, N. Y.; John C. Lewis, Dewittville. N. Y.; John Adelbert Love, Stockton, N. Y.; William Chancellor R[illegible]ney, Wright, N. Y. David Benjamin Horton, Wolcott. N. Y.; Henry Martin Bishop, Edwardsburgh, Mich.; Daniel Curtis, St. Thomas, Ont.; James Monr e Callender, Scranton, Pa.; George Washington Pearson, Nippinose, Pa.; Clinton Allen Sage, Pekin, N. Y.; Frank D. Parker, Akron, N. Y.; Thomas F. Major, Hornellsville, N. Y.; Ransom Terry, Ischua, N. Y.; Marcenos Henry Cole, Pittsford, N. Y.; Adin Pattin Waid, Centreville, Pa.; Robert H. Hewes, Western, N. Y.

He also announced that the Faculty and Curators deem a thesis upon "Syphilitic Affections of the Nervous System," by Edward N. Brush, and one entitled, "To what are the Changes in the Color of the Blood due?" By Edward Mott Moore, Jr., worthy of honorable mention and recommend the same for publication; also a thesis by Bernard Bartow upon Aneurism, worthy of honorable mention. After music by the orchestra, the Diplomas were delivered to the class by Prof. James P White, President, *pro tem*, of the Council; this was followed by music after which Mr. Joseph Warren was introduced to the audience and delivered the address to the graduating class. The address was listened to with marked attention and frequently called forth hearty applause. Want of space forbids our publishing it in this issue of the JOURNAL, it will however appear in the March number. Music and the benediction by Rev. Mr. Witherspoon closed the exercises.

